# Music Does Not Facilitate Lexical Tone Normalization: A Speech-Specific Perceptual Process

**DOI:** 10.3389/fpsyg.2021.717110

**Published:** 2021-10-22

**Authors:** Ran Tao, Kaile Zhang, Gang Peng

**Affiliations:** Research Centre for Language, Cognition, and Neuroscience, Department of Chinese and Bilingual Studies, The Hong Kong Polytechnic University, Kowloon, Hong Kong SAR, China

**Keywords:** speech normalization, tone normalization, music, lexical tones, Cantonese

## Abstract

Listeners utilize the immediate contexts to efficiently normalize variable vocal streams into standard phonology units. However, researchers debated whether non-speech contexts can also serve as valid clues for speech normalization. Supporters of the two sides proposed a general-auditory hypothesis and a speech-specific hypothesis to explain the underlying mechanisms. A possible confounding factor of this inconsistency is the listeners’ perceptual familiarity of the contexts, as the non-speech contexts were perceptually unfamiliar to listeners. In this study, we examined this confounding factor by recruiting a group of native Cantonese speakers with sufficient musical training experience and a control group with minimal musical training. Participants performed lexical tone judgment tasks in three contextual conditions, i.e., speech, non-speech, and music context conditions. Both groups were familiar with the speech context and not familiar with the non-speech context. The musician group was more familiar with the music context than the non-musician group. The results evidenced the lexical tone normalization process in speech context but not non-speech nor music contexts. More importantly, musicians did not outperform non-musicians on any contextual conditions even if the musicians were experienced at pitch perception, indicating that there is no noticeable transfer in pitch perception from the music domain to the linguistic domain for tonal language speakers. The findings showed that even high familiarity with a non-linguistic context cannot elicit an effective lexical tone normalization process, supporting the speech-specific basis of the perceptual normalization process.

## Introduction

Humans communicate in language and music. In both formats, the continuous acoustic signals are segmented and then categorized into abstract meaningful units (e.g., words and melodies). Musical performance and appreciation require deliberate practice and longitudinal exposure, but speech production and perception abilities are developed naturally. Albeit speech categorization is sometimes demanding since there is no one-to-one mapping between acoustic signals and linguistic units due to speaker variability. In speech production, speakers vary a lot in their vocal tract configurations, which results in a large individual difference in speech production (Peterson and Barney, 1952). Even speech production by the same speaker may change a lot in different situations (Newman et al., 2001). The inter- and intra-speaker variability blurs the boundary between two acoustically similar phonemes and makes them less distinguishable. For example, a male speaker’s production of a high-level tone may have a similar pitch height as a female speaker’s production of a mid-level tone (Peng et al., 2012). The word identification is slower and less accurate when the speech stimuli are presented in the mixed-speaker condition than in the blocked-talker condition (Nusbaum and Magnuson, 1997; Magnuson and Nusbaum, 2007), revealing an obstacle in speech perception introduced by high speech variability.

Language as a complex system also provided rich information for us to overcome the speech variability and achieve perceptual constancy. Ladefoged and Broadbent (1957) found that the ambiguous ‘bVt’ syllable was more likely perceived as ‘bit’ in a sentence with high first formant (F1) and as ‘bet’ in a sentence with low F1. This pioneering work demonstrates that the acoustic information embedded in context affects our interpretation of the target speech cues and thus to some extent reduces the ambiguity caused by the inter- and intra-talker variability, a process known as extrinsic normalization (Nearey, 1989). The extrinsic normalization has been widely observed in the perception of vowels, consonants, and lexical tones. The perception of Cantonese lexical tones relies heavily on the extrinsic context information. The primary acoustic correlate of lexical tones is the fundamental frequency (F0), and the height and the slope of F0 trajectory affect the tone differentiation (Gandour, 1983). However, Cantonese has three level tones, high-level tone, middle-level tone, and low-level tone, which can only be differentiated by pitch heights (Peng, 2006). Therefore, the contextual F0 which provides a good reference for listeners to estimate the relative pitch height of the target stimuli becomes important. Cantonese speakers’ perception of three level tones was improved significantly (from 48.6% to above 90%) when the isolated tonal stimuli were embedded into speech contexts (Wong and Diehl, 2003). Wong and Diehl (2003) and Zhang et al. (2013,^2017^,^2021^) reported that an ambiguous Cantonese middle-level tone is more frequently perceived as the high-level tone after a context of low F0 and perceived as the low-level tone after a context of high F0, indicating a contrastive context effect in the lexical tone normalization process.

### Spectrally Contrastive Encoding and General-Auditory Level Processing

Since the observation of context effect, different theories are proposed to explain the underlying mechanisms of the extrinsic normalization process. There is an ongoing dispute about whether the extrinsic normalization operates on the general-auditory level or the speech-specific level. The core issue of this debate mainly lies in the effectiveness of non-speech context on perceptual normalization. Huang and Holt (2009) reported that Mandarin lexical tone perception was contrastively affected by the non-speech context composed of the sine-wave harmonics or the pure tone. Specifically, listeners perceived the ambiguous lexical tones as high-level tone (Mandarin T1) if its preceding non-speech context had low frequency and as high-rising tone (Mandarin T2) if the preceding non-speech context had high frequency. More importantly, the contrastive context effect in non-speech contexts (albeit quantitatively smaller) was statistically comparable with that in the speech context. The effectiveness of non-speech contexts got strong support from a serial of studies on consonant normalization as well. The non-speech composed of sine-wave tones and white noise affected listeners’ perception of /ga/-/da/continuum in a similar manner as the speech context (Lotto and Kluender, 1998; Holt, 2006), even if context and target were separated by more than one second or multiple intervening sounds (Holt, 2005). Japanese quails demonstrated the contrastive perception behavior after training, which further suggested that the normalization process was not constrained by the speech-specific processing (Lotto et al., 1997). Since non-speech is extralinguistic, Lotto and Kluender (1998) suggested that the normalization process required no specific linguistic knowledge and that it operated on the general perceptual level and depended on the spectral contrast between context and target stimuli.

The contrastive perceptual pattern is essentially consistent with forwarding energy masking, which shows that the target after the masker is perceived less accurately when the masker has acoustic energies in the same frequency region as the target (Moore, 1995; Viswanathan et al., 2013). Energy masking is partially caused by the inertia of the auditory nerve; that is, the basilar membrane takes time to recover after responding to the masker (Duifhuis, 1973). Aside from the physiological basis in the peripheral auditory system, the neural adaptation of the central auditory system also contributes to contrast encoding (e.g., Bertrand, 1997). As illustrated by the oddball paradigm, while being continuously exposed to the same auditory stimuli, neurons decrease their firing rates and become less active; when new stimuli are presented, neurons are activated and increase their firing rates, generating large event-related potential (ERP) amplitude (Polich, 2007). This working mechanism of neurons may also apply to the extrinsic normalization process. Neurons in the auditory cortex are responsive to different frequency regions (Sjerps et al., 2019). Neurons adapted by the preceding context are less responsive to the same frequencies in the following target, but neurons that do not fire in the context presentation are relatively more sensitive to the frequency ranges of the following sounds, resulting in spectrally contrastive perception (Stilp, 2020).

### Context Tuning and Speech-Specific Level Processing

The normalization differences between speech and non-speech contexts were also reported. Francis et al. (2006) compared the normalization of Cantonese middle-level tones in speech and non-speech contexts. The speech context was meaningful Cantonese sentence /ŋƆ23 wui23 tƆk22 ji33 pƐi25 lƐi23 t’Ɛŋ55/[I will read ji for you (to hear)], and the non-speech context was synthesized by applying the pitch contour of the speech context to the ‘hummed’ unintelligible neutral vocal tract /ǝ/ with Praat (Boersma and Weenink, 2016). They found that even though the non-speech context contained the crucial cues for the pitch range estimation (i.e., the same pitch contour as the speech context), native listeners showed almost no normalization effect in the non-speech context. However, the contrastive perceptual pattern was noticeable in the speech context. Their study revealed an unequal effect of speech and non-speech contexts in the lexical tone normalization process. Zhang et al. (2015) further tested the contribution of speech information at each level. They asked native Cantonese speakers to identify the ambiguous Cantonese middle-level tones in non-speech contexts (triangle waves), reversed speech contexts (normal speech reversed in time scale, sounding like foreign phonemes), meaningless speech contexts (Cantonese monosyllabic sequences), and meaningful speech contexts. The information in four contexts also decreased from meaningful speech (semantic, phonological, phonetic, and acoustic information), meaningless speech (phonological, phonetic, and acoustic information), reversed speech (phonetic and acoustic information), to non-speech (acoustic information). They found that meaningful speech exerted the largest normalization effect, which was followed by the meaningless speech contexts. The reversed speech context also showed some positive effects on the normalization process, but the normalization effect in non-speech was almost negligible. Francis et al. (2006) and Zhang et al. (2015) suggest that the normalization effect of non-speech context is not as prominent as speech context and that speech-specific information (i.e., semantic, phonological, and phonetic information) is necessary for the Cantonese level tone normalization.

Instead of a contrastive encoding of auditory signals, some researchers believe that the speech normalization process operates via the context tuning mechanism. According to the context tuning mechanism, listeners use extrinsic contextual information to compute a talker-specific mapping of acoustic patterns onto abstract linguistic units, and ambiguous target speech cues are identified by referring to that mapping. Joos (1948) described that a talker-specific vowel pattern can be quickly established even during their first greeting ‘How do you do?’. Three critical phonemes /a/, /j/, and /u/in the greeting can roughly outline the vowel space of that speaker since the /a/ is pronounced with the low central articulation gesture, the /u/ with the highest and strongest back articulation, and the /j/ with a higher and more forward articulation. The incoming acoustic signals can be categorized by referring to this vowel pattern. Joos’ description indicates that the mapping used in normalization is essentially an acoustic-phoneme mapping. To form such a mapping, linguistic knowledge is required, indicating a normalization process at the speech-specific level.

### A Hybrid Model of Speech Perception: Co-existence of Exemplars and Abstract Linguistic Representations

Although general auditory contrastive encoding mechanism and the context tuning mechanism, to some extent, explain the context effect on ambiguous speech perception, they can hardly explain why the typical speech is almost not affected by context cues. For example, the perception of two endpoints of the speech continuum does not change in different context shifts (Johnson, 1990) and speakers whose pitch ranges are closer to the population mean are less affected by the context F0 as well (Zhang et al., 2013). These findings suggest that while perceiving speech in context, contextual cues only partially contribute to our final decision and that another ongoing perceptual mechanism that utilizes the specific characteristics of each token also affects our final phonemic categorization. The token-specific effect can be well explained by the exemplar-based theory which believes that the exemplars we encounter in our daily life form the mental representations of each phonological category, and that speech perception is a match between stored exemplars and the incoming signal (Johnson, 1997). Based on this account, the speaker-specific details are also kept with the exemplars and are helpful cues for speech perception.

The normalization approach emphasizes the computation of an abstract and speaker-independent mental representation, but the exemplar-based approach utilizes the speaker-specific details to match the stored exemplars. Considering that both abstract phonological categories and fine acoustic details were reported to affect speech perception, a hybrid model was proposed to accommodate these two different views (Tuller, 2003; Nguyen et al., 2009). In the hybrid model, the mental representation of each phonological category is a multi-layered construct. Listeners maintain multiple exemplars with speaker-specific details in the lower layer and these exemplars gradually decay into more abstract speaker-independent representations in the upper layer. Correspondingly, speech perception may be a multi-pathway process as well. The normalization process extracts invariant elements from speech tokens and matches them to the abstract phonological categories. Meanwhile, the details of speech tokens are kept in memory, which constrains speech categorization.

To investigate the mental representation of phonological units, Kimball et al. (2015) asked listeners to identify whether two sounds in a trial were the same or different. The different trial was either the phonological variation (presence or absence of a pitch accent) or the phonetic variation (different durations or F0 peaks). They found that listeners could accurately identify the trials differing either in the phonological level or the phonetic level. When the interval between two sounds increased, the accuracy dropped for the phonetic variation but not for the phonological variation. Their results suggested that both phonetic details and abstract phonological categories were kept in our memory, and phonological distinctions were more robust, supporting the hybrid model of speech perception.

### Familiarity and the Speech Normalization Process

The hybrid model, especially the exemplar layer, challenges the account that the speech superiority is due to the speech-specific nature of the normalization process. Although previous research made spectral complexity and spectral contrast comparable in speech and non-speech contexts, speech and non-speech still differ in many aspects. In addition to the speech-specific information (i.e., phonetic, phonological, and semantic information) which may favor a speech-specific account, listeners have different familiarities with speech and non-speech stimuli. Compared with the non-speech contexts used in previous studies (e.g., pure tones, harmonic complex tones, triangular waves, hummed sound modeled on a neutral vocal tract, or iterated rippled noise), listeners are much more familiar with speech contexts. They store more exemplars of speech than non-speech in their daily exposure to sound, and robust phonemic representations are established during their long-term language acquisition and usage. The rich exemplars on the lower layer and the robust phonemic representation on the higher layer result in a stronger activation of speech context than non-speech context. Familiarity also affects the efficiency of speech perception, which is boosted due to the countless repetition in daily communication, but the decoding of non-speech is rare and thus less automatic. The spectral characteristics in the non-speech context are probably not utilized due to the weak activation and/or limited process.

Familiarity advantage has been widely reported in speech perception studies, for example, faster response to speech spoken in familiar languages (e.g., Hu et al., 2017) and better identification of familiar talkers’ speech in noise (e.g., Nygaard and Pisoni, 1998). Familiarity advantage might exist in the perceptual normalization as well since previous studies found that listeners’ speech perception is better with contexts spoken in their native language (e.g., Lee et al., 2009; Kang et al., 2016; Zhang, 2020). Lee et al. (2009) asked native Mandarin speakers and native English speakers to identify the digitally processed Mandarin tones either in isolation or with Mandarin context. These syllables were produced by either single talker or multiple talkers. Talker variability affected Mandarin and English listeners equally. However, Mandarin listeners made better use of context to compensate for the speech variability and improved the lexical tone identification, suggesting a language familiarity advantage in the extrinsic normalization of lexical tones. Similar results were also reported in the extrinsic normalization of segmental components. The context composed of vowel /y/ can facilitate French speakers but not English speakers to perceive ambiguous /s–∫/ sound probably because vowel /y/ exists in French but not in English (Kang et al., 2016). The context effect of the native context /ε i/ was more prominent than that of the non-native context /oe y/ when English speakers perceived ambiguous vowel /u–Ɔ/ (Zhang, 2020).

However, different findings were also reported. Sjerps and Smiljanić (2013) tested how language familiarity affected vowel normalization. They asked native listeners of American English, Dutch, Spanish and Spanish-English bilinguals to perceive the ambiguous /sofo/-/sufu/ with contexts spoken in either Dutch, Spanish, or English. They found that the perceptual impact of precursor context was comparable in size across listeners’ language backgrounds, indicating a weak effect of the language familiarity on the speech normalization process. Magnuson et al. (2021) tested how the talker familiarity affected the accommodation of speech variabilities. They asked native Japanese speakers to identify morae produced by either familiar talkers (family members) or strangers in either blocked- or mixed-talker conditions. Listeners always took a longer time to recognize morae in the mixed-talker condition even for the voice of their family members, indicating a constant cost for talker accommodation regardless of talker familiarity.

Albeit inconclusive, the familiarity difference between speech and non-speech material is a potential factor that contributes to the superiority of speech context in the normalization process. Probably, either familiarity or speech-specific information contributes to the normalization process, or they work together to facilitate speech normalization. By teasing familiarity and speech-specific information apart, we can test if the speech-specific information is the only or main contributor to the speech-superiority effect, which may partially clarify the long-standing dispute between the general-auditory and speech-specific basis of the extrinsic lexical tone normalization. If normalization effects are comparable between speech and non-speech contexts when the familiarity gap is controlled, the speech-specific information might not play a crucial role in extrinsic normalization and the normalization process is largely processed by a general-auditory mechanism. However, if speech context still shows a significantly better normalization effect than other contexts when their familiarities are comparable, this could be strong evidence for the speech-specific basis of extrinsic normalization.

To test the confounding factor familiarity, the present study compares the native Cantonese speakers’ perception of ambiguous Cantonese level tones in the context of either speech, music, or synthesized non-speech. Music is one of the most meaningful and popular forms of non-verbal sound; like speech, it has been developed to take advantage of the efficiencies of the human auditory system (Baldwin, 2012). Music also follows syntax-like rules, makes use of pitch and rhythm, and has a ubiquitous presence across human civilizations (Zatorre et al., 2007; Patel, 2013). The prevalence and the use of pitch as an importation cue make music an ideal non-speech context for the present study to test the familiarity effect. For Cantonese speakers who never received professional musical training, their familiarities with the three contexts decrease from speech, music, to synthesized non-speech. Meanwhile, familiarity is further manipulated by including a group of Cantonese-speaking musicians who receive professional musical training and thus are much more familiar with musical materials than non-musicians. If familiarity is the main factor for the unequal effect of the speech and non-speech context, musicians are expected to perform better than non-musicians at least in the musical context, and meanwhile, both groups are expected to show a performance improvement when contexts change from synthesized non-speech, music to speech.

### Music Experience and the Lexical Tone Normalization Process

To our knowledge, no study directly tested how music experience affects the lexical tone normalization. A few studies about the congenital amusia, a neurodevelopmental disorder of pitch processing (Ayotte et al., 2002) may shade light on this question from a different angle, that is how the music deficit affects the lexical tone normalization. People with congenital amusia (amusics) showed severe deficit in the perception of musical melody. Zhang C. et al. (2018) asked Cantonese-speaking amusics and controls to perceive the ambiguous Cantonese mid-level tone in contexts with different pitch heights. The control group showed noticeable normalization effect in speech context, but the normalization effect in speech context is much reduced for amusics. Shao and Zhang (2018) further tested the perception of six Cantonese tones with and without context for two groups. They found that controls performed better with context cues, but that amusics in most cases failed to benefit from the context cues. Similar result was also observed in Mandarin speakers. Liu et al. (2021) reported that Mandarin-speaking amusics cannot utilize the contextual information to perceive the ambiguous Mandarin T55–T35 continuum, but control group without amusia showed typical context effect in the Mandarin lexical tone perception. The studies about amusia suggest that the impaired pitch perception ability in music domain affects the lexical tone normalization. Based on the findings from amusics, it is natural to hypothesize that people with music experience probably perform better in the lexical tone normalization.

This hypothesis is somewhat supported by the studies which reported that music experience affects lexical tone perception (for a review, please see Ong et al., 2020). While detecting the subtle sentence-final pitch variation, French musicians performed better than non-musicians no matter in their native language (Schön et al., 2004) or the non-native language (Marques et al., 2007). French musicians could also detect the variation of Mandarin lexical tones better than non-musicians (Marie et al., 2011). English Musicians’ identification and discrimination of Mandarin lexical tones were faster and more accurate than non-musicians (Alexander et al., 2005). Even musicians of tonal language speakers, for example, Mandarin musicians, showed increased sensitivity to the fine acoustic difference of mandarin tones (Wu et al., 2015). All these findings support a positive transfer from music experience to pitch perception in the language domain. Considering musicians’ improved pitch perception ability, they are expected to extract the contextual pitch information more accurately and thus have a more precise pitch range reference to estimate the relative pitch height of the target tone. Therefore, these tone perception studies make it more reasonable to hypothesize that music experience boosts the extrinsic normalization of lexical tones.

The present study also explicitly tests this hypothesis by comparing musicians who receive intensive and professional music training with non-musicians who have rare music experience in a lexical tone normalization task. If the hypothesis holds, musicians are expected to show a stronger context effect than non-musicians at least in the speech context condition. Considering that musicians are reported to have better pitch perception ability in both linguistic and non-linguistic domains, they probably perform better than non-musicians in the non-speech contexts (music and synthesized non-speech) as well. If this is true, the extrinsic normalization of lexical tones is largely determined by the domain-general pitch processing ability but not the speech-specific processing, which to some extent is in line with the general auditory mechanism and the familiarity hypothesis (i.e., the frequent practice in pitch perception). On the contrary, if musicians fail to show any advantage over non-musicians in tone normalization in any kind of context conditions, the results will favor a speech-specific mechanism.

## Materials and Methods

### Participants

Forty native Cantonese adults participated in this experiment, among whom 20 were categorized as non-musicians (10 female, Age = 21.9 ± 2.96) and 20 were categorized as musicians (10 female, Age = 23.6 ± 4.69). Participants were matched in their age [Welch’s *t*(32.1) = 1.325, *p* = 0.194] and gender. Non-musicians were defined as individuals with less than 3 years of musical training except the mandatory courses in their primary or middle schools. Musicians were defined as individuals with at least 7 years of private musical training and still actively engaging in music (Wong et al., 2007; Wayland et al., 2010; Cooper and Wang, 2012), such as practicing music, studying in music major, or having a music-related occupation (e.g., band member, private music tutor, and music teacher in schools). The musicians had a diverse background of music learning experience and some of them reported having learned several kinds of instruments. Characteristics of musicians are summarized in the Supplementary Table S1. One non-musician was identified as ambidextrous using the Edinburgh handedness inventory (Oldfield, 1971) and the rest participants were right-handed. All participants reported no hearing loss, neuropsychiatric disorders, or brain injuries. Participants were compensated for their time and signed consent forms before the experiment. The experiment procedure was approved by the Human Subjects Ethics Sub-committee of The Hong Kong Polytechnic University.

### Stimuli

Preparation of the stimuli and the experimental procedure followed previous work (Zhang et al., 2013, 2017; Tao and Peng, 2020). Stimuli consisted of contexts and targets in four different context conditions: a speech context condition, two non-linguistic contexts (e.g., synthesized non-speech and music), and a condition without context (coded as isolated hereafter). Speech contexts and all targets were produced by four native Cantonese talkers who were a female talker with a high pitch range, a female talker with a low pitch range, a male talker with a high pitch range, and a male talker with a low pitch range (coded as FH, FL, MH, and ML respectively). Speech context was a four-syllable meaningful sentence, i.e., 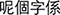 (/li55 ko33 tsi22 h
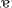
i22/, “This word is meaning”). After recording the natural production of the sentence from the four talkers, the F0 trajectories of the sentences (see Supplementary Figure S1) were then lowered and raised three semitones to trigger the contrastive context effect (Wong and Diehl, 2003). Specifically, more high-level tone responses were expected in the lowered context condition and more low-level tone responses in the raised context condition. In sum, three sets of speech contexts were formed: a set of F0 lowered contexts, a set of F0 unshifted contexts, and a set of F0 raised contexts. All targets from four context conditions were the natural production of the Chinese character 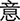 (e.g., /ji33/mid-level tone, “meaning,” also see Supplementary Figure S1 for F0 trajectories).

**FIGURE 1:**
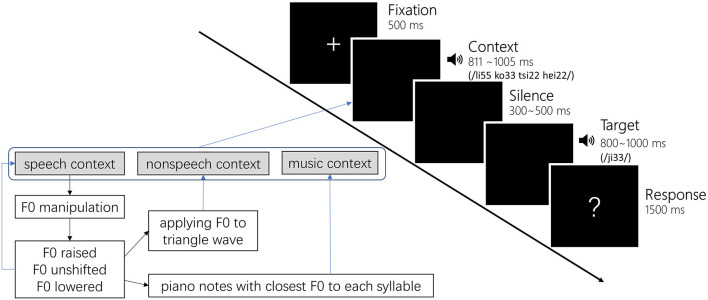
Trial procedure of the word identification task and a schema depicting the context preparation.

The non-speech contexts were produced by applying the F0 trajectory and intensity profile from speech contexts to triangle waves. The music contexts were piano notes that had the closest pitch height to each of the syllables in the speech context, which were generated using a Kurzweil K2000 synthesizer tuned to the standard A4 of 440 Hz (Peng et al., 2013). We chose the closest piano notes rather than synthesizing a piano sound with the mean F0 of each syllable to ensure that the musicians would feel as natural as possible when hearing these notes. The manipulation on speech F0 and selection of piano notes caused a slight discrepancy between conditions (see Table 1 for a list of mean F0 of all contexts and Targets), however, the hierarchy of F0 between raised, unshifted, and lowered conditions were reliably reserved. Figure 1 lower panel shows a schema of context stimuli preparation. All speech stimuli, including speech contexts and targets, were adjusted to 55 dB in intensity. The non-linguistic contexts, including non-speech and music contexts, were adjusted to 75 dB in intensity to match the hearing loudness of speech contexts. The duration of speech contexts was kept unchanged to reserve the natural production outcome (FH: 1005 ms, FL: 888 ms, MH: 811 ms, ML: 821 ms). The duration of non-speech contexts was the same as their corresponding speech contexts. The duration of music contexts was 1000 ms with each note lasting 250 ms.

**TABLE 1:** Mean fundamental frequency (Hz) of speech contexts, their counterparts, and targets.

		FH	FL	MH	ML
Speech/non-speech	F0 raised	280.5	246.3	174.6	134.5
	F0 unshifted	236.8	208.1	148.4	113.8
	F0 lowered	198.2	173.9	124.5	96.8
Music	F0 raised	294.4	255.6	181.6	139.6
	F0 unshifted	247.7	215.4	153.8	117.6
	F0 lowered	207.6	181.2	128.7	98.7
Target		233.2	206.8	143.8	114.9

Fillers were prepared with the same procedure. In the speech context condition, the filling context was two four-syllable sentences, i.e., 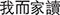 (/ŋo23 ji21 ka55 tuk2/, “Now I will read,” recorded from FL and MH) and 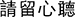 (/ts^h^iŋ25 lǝu21 sm55 t^h^iŋ55/, “Please listen carefully to,” recorded from FH and ML). Target fillers were Chinese characters 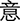 (recorded from FL and MH) or 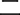 (e.g., /ji22/ low-level tone, “two,” recorded from FH and ML).

### Experiment Procedure

All participants attended a word identification task in a sound-proof booth. Participants were asked to make a judgment on the target syllable following a preceding context. In each experiment trial, the target and context corresponded with each other, i.e., the target always followed the context produced by the same talker or its non-verbal counterparts. Participants were instructed to listen to both the context and the target attentively. Specifically, they first saw a 500 ms fixation in the middle of the screen followed by the context presented through earplugs, and then after a jittering silence (range: 300–500 ms), a target syllable was presented. In the isolated condition, participants heard the target without a context, i.e., the fixation was followed by the jittering silence immediately. Participants then made a judgment on the target syllable from three choices of 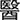 (/ji55/ high-level tone, “doctor”), 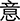 (middle-level tone), or 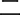 (low-level tone) by pressing designated keys on the keyboard when they saw a cue on the screen. The cue was a question mark on the middle of the screen, delayed 800–1000 ms from the onset of the target (see Figure 1). In this kind of setting, reaction times were not meaningful indices of participants’ psycholinguistic processing and thus were not analyzed in this study. We focused on the participants’ judgments on the targets.

The four context conditions were grouped into four experimental blocks which were counterbalanced across participants to prevent order effect. The isolated condition block consisted of 16 repetitions of each target. The blocks of three context conditions each consisted of nine repetitions of three F0 shifts of four talkers.

### Analysis

First, we evaluated the effect of a preceding context on the perception of middle-level tones by comparing the listeners’ response patterns on the targets following various contexts (and without a context, e.g., isolated condition) with a three-way ANOVA. Two within-subject factors were Context (isolated, music, non-speech, speech) and Choice (judging the target as high-, middle-, and low-level tones), and one between-subject factor was Group (musicians and non-musicians). In this analysis, we included the isolated condition and three context conditions in which the contexts’ F0 were kept unshifted (F0 unshifted context conditions), such that the targets’ F0 fell in the range of contexts’ F0. This analysis revealed whether the perception of Cantonese middle-level tone was regulated by preceding contexts. We were particularly interested in the comparison between the isolated condition and the other three context conditions as the isolated condition served as a benchmark indicating the response bias toward a middle-level tone when the target presented individually. According to Wong and Diehl (2003), the response rate of middle-level tone in the isolated condition could be around 50% across talkers. The context condition eliciting a different response rate than the isolated condition should inform us that the context provided useful information for listeners to adopt in normalizing the level tone. However, a lack of difference in F0 unshifted context conditions might not conclude that the context failed to support listeners’ lexical tone normalization, so a second analysis including the various F0 manipulations was performed, which focused on the contrastive context effect.

Following previous research on contrastive context effect (Wong and Diehl, 2003; Zhang et al., 2012, 2017), perceptual height (PH) and expected identification rate (IR) were analyzed to investigate participants’ lexical tone normalization performance. For the PH analysis, a response of high-level tone was coded as 6, middle-level tone as 3, and low-level tone as 1. This coding scheme reflected the acoustic difference among the three level tones and was straightforward when deciphering the results. The mean PH close to 6 indicated that participants generally perceived the targets as high-level tones. In an F0 lowered condition, this could serve as evidence of evoking participants’ tone normalization. The mean PH close to 1 indicated that participants generally perceived the targets as low-level tone. In an F0 raised condition, this could serve as evidence of evoking participants’ tone normalization. The IR was the percentage of expected responses in each condition according to the contrastive context effect. The expected responses were the judgments that participants should make when the lexical tone normalization process was elicited, e.g., choosing low-level tone in the F0 raised condition, and choosing high-level tone in the F0 lowered condition. For targets following an F0 unshifted context, although there is no contrast between the context and target, it is expected that participants would perceive the target as a middle-level tone if the context provides sufficient information to reliably categorize the level tone.

We conducted three-way ANOVAs on PH and IR, where the isolated condition was excluded as it did not match the design matrix of other context conditions, e.g., there was no context and thus no F0 Shift manipulations. Two within-subject factors were Context (music, non-speech, speech) and Shift (F0 lowered, unshifted, raised), and one between-subject factor was Group (musicians, non-musicians). It is expected to see a contrastive context effect in speech context conditions and a lack of such an effect in non-speech context conditions. Following a speech-specific mechanism hypothesis, the music context conditions would not elicit a contrastive context effect, while the general-auditory mechanism hypothesis would expect music context to elicit a contrastive context effect, with higher magnitude seen in the musician group, e.g., an interaction between the three factors. The interaction among Context, Shift, and Group factors was most critical to the current study.

Previous research suggested that speech contexts produced by talkers with various F0 all elicited very high IR, while the specific pattern of responses was biased by the talkers’ F0 (Zhang et al., 2013). For example, both female and male talkers with lower F0 elicited more low-level tone responses, and both female and male talkers with higher F0 elicited more high-level tone responses. The present study did not aim to follow up the discussion on the talker effect, nonetheless, the four talkers were included in the experiment to prevent response bias to a single talker and to increase the generalization of the results. Two talker-related factors, Gender (with two levels, female and male) and Pitch (with two levels, high pitch and low pitch) were included as control variables in all analyses for controlling their main effects and possible interactions with other factors (but see Supplementary Figures S2, S3 for the illustrations of talker effects on PH and IR).

In all analyses, Greenhouse–Geisser correction was applied when the data violated the Sphericity hypothesis. Tukey method for comparing families of multiple estimates was applied for necessary *post hoc* analysis. The effect size of each significant main effect and interaction was reported in the form of general eta squared (η^2^). The ANOVA procedure is robust for within-subject designs and used for analyzing data in this study to increase the comparability with previous research. However, we also performed a non-parametric version of ANOVA and found the results were highly similar (see Supplementary Analysis). All analysis was performed in R (version 4.0.5, R Core Team, 2021) with packages tidyverse (Wickham et al., 2019), rstatix (Kassambara, 2021), afex (Singmann et al., 2021), lsmeans (Lenth, 2016), and ggplot2 (Wickham, 2016) for data processing, statistics, and visualization.

## Results

### Context Regulation on Targets’ Response Rates

**FIGURE 2:**
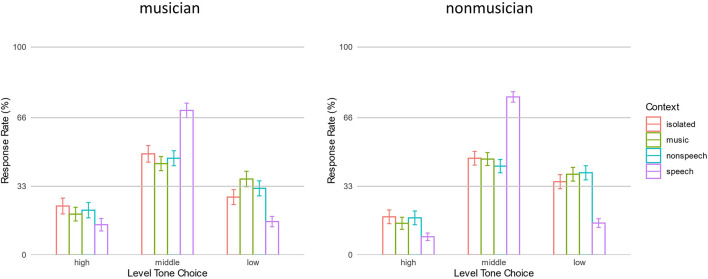
The response rates of high, middle, and low-level tones in isolated and three context conditions. Error bars represent standard error.

To evaluate how the context regulated participants’ response rates on the three possible choices (high, middle, and low-level tones), ANOVA was performed on target response rates of F0 unshifted context conditions and isolated condition. Results revealed a main effect of Choice [*F*(1.84,70.08) = 54.69, *p* < 0.001, η^2^ = 0.226]. The response rate of middle-level tone (mean ± SD = 52.5% ± 12.5%) was higher than other responses (*p*s < 0.001), and response rate of low-level tone (30.1% ± 10.7%) was higher than high-level tone (17.7% ± 13.8%). There was an interaction between Context and Choice factors [*F*(4.64, 176.47) = 26.54, *p* < 0.001, η^2^ = 0.097]. As in Figure 2, *post hoc* analysis revealed that the response rates of all three level tones following speech contexts were different from the isolated condition. The speech context yielded a higher response rate of middle-level tone than isolated condition (72.7% ± 21.2% vs. 47.6% ± 17.1%, *p* < 0.001), and lower responses rates of high- and low-level tones (11.6% ± 15.2% vs. 20.9% ± 19.4%, *p* < 0.01 and 15.7% ± 12.1% vs. 31.5% ± 14.4%, *p* < 0.001, respectively). The music and non-speech contexts, however, did not yield different response rates from isolated conditions (all *p*s > 0.1). The Group factor did not interact with other factors (*p*s > 0.4), suggesting that the above pattern was consistent across musicians and non-musicians. The results indicated that a speech context could regulate listeners’ responses to targets: listeners had a higher rate of making the correct choice, i.e., choosing middle-level tone.

### Extrinsic Normalization in Three Context Conditions

The lack of response rate differences from isolated conditions suggested that non-speech and music context could not facilitate tone categorization when the target F0 fell in the context’s F0 range. However, the results could not conclude that non-speech and music context fail to facilitate tone categorization in a contrastive manner. Therefore, ANOVA was also performed on PH and IR, respectively. The isolated context condition was excluded from these analyses for not matching the other three context conditions on the Shift factor and not possible to elicit contrastive extrinsic normalization, e.g., there are no contexts and thus no F0 manipulations.

For the analysis on PH, a main effect of Context [*F*(1.99,75.49) = 13.32, *p* < 0.001, η^2^ = 0.040] was found, with speech context yielded higher PH than non-speech and music contexts (*p*s < 0.01). There was no difference between PH yielded by non-speech and music contexts (*p* = 0.341). Additionally, a main effect of Shift was found [*F*(1.21,46.13) = 240.46, *p* < 0.001, η^2^ = 0.181]. *Post hoc* analysis revealed that F0 lowered contexts yielded a higher PH than F0 unshifted contexts which was higher than F0 raised contexts (lower > unshifted > raised: 3.62 ± 0.61 > 2.89 ± 0.53 > 2.36 ± 0.58, all *p*s < 0.001). Not surprisingly, there was an interaction between the Context and Shift factors [*F*(1.44, 54.80) = 216.33, *p* < 0.001, η^2^ = 0.307]. However, the decremental pattern of F0 manipulation (lowered > unshifted > raised) was only significant in speech contexts (5.28 ± 1.00 > 3.04 ± 0.47 > 1.48 ± 0.69, *p*s < 0.001), but not non-speech (2.90 ± 0.78, 2.88 ± 0.75, 2.87 ± 0.812) nor music (2.69 ± 0.76, 2.77 ± 0.74, 2.72 ± 0.76, all *p*s > 0.7) context conditions. The decrement of PH with F0 manipulation in speech context conditions is evident for the contrastive context effect, and thus indicate that listeners were elicited lexical tone normalization in the speech context conditions only but not in non-speech nor music context conditions.

**FIGURE 3:**
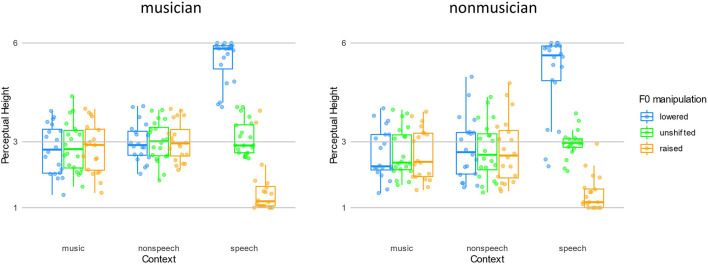
The perceptual height of the targets following three contexts with F0 manipulations in two groups.

It is also worth mentioning that the main effect of Group was not significant [*F*(1,38) = 1.59, *p* = 0.214, η^2^ = 0.009]. Group factor did not interact with Context (*p* = 0.928) or Shift factors (*p* = 0.879), and there was not a three-way interaction among these factors (*p* = 0.572). Such a pattern indicated that musicians did not outperform non-musicians in any of the contexts with any kind of F0 manipulations (Figure 3).

**FIGURE 4:**
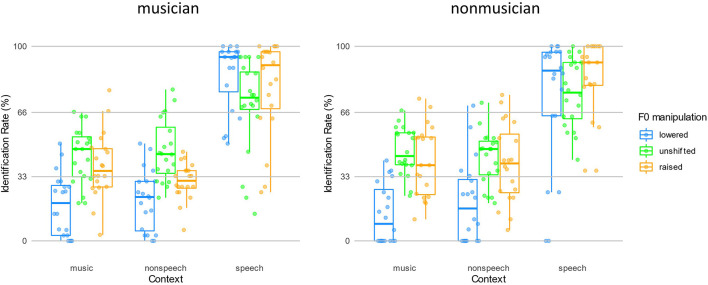
The identification rate of the target following three contexts with F0 manipulations in two groups.

The analysis on PH revealed that participants perceived targets as perceptually different tones only in speech contexts in both groups. To test whether participants behaved categorically following expectations of contrastive context effect, we performed ANOVA on their IR synthesized across each condition. The ANOVA on IR found a main effect of Context [*F*(1.10,41.68) = 198.90, *p* < 0.001, η^2^ = 0.365], driven by that the speech context yielded a higher IR than non-speech and music contexts (*p*s < 0.001). The main effect of Shift was also significant [*F*(1.67, 63.38) = 13.89, *p* < 0.001, η^2^ = 0.058]. The *post hoc* analysis revealed that both F0 unshifted (54.1% ± 12.4%) and raised (52.0% ± 12.8%) conditions yielded higher IR than F0 lower condition (38.7% ± 14.7%) (*p*s < 0.001), while the F0 unshifted and raised conditions yielded similar IR (*p* = 0.794). There was also an interaction between the Context and Shift factors [*F*(3.47, 132.00) = 18.75, *p* < 0.001, η^2^ = 0.054], which was driven by the similarly high IRs yielded by F0 manipulations in the speech context conditions (lowered, unshifted, raised: 79.5% ± 27.1%, 72.7% ± 21.2%, 81.2% ± 22.2%, all *p*s > 0.1) and different but low IRs observed in non-speech and music contexts. In both non-speech and music contexts, the patterns of IRs yielded by F0 manipulations were similar: the F0 lowered (in non-speech and music: 20.4% ± 18.2% and 16.1% ± 15.6%) condition yielded a smaller IR than unshifted (44.6% ± 14.9% and 45.0% ± 13.3%) and raised (35.2% ± 16.3% and 39.7% ± 17.5%) conditions (all *p*s < 0.001), and the unshifted and raised conditions yielded similar IRs (*p*s > 0.05). The similarly high IRs observed in speech context with F0 manipulations indicated that listeners’ performance followed the contrastive context effect’s expectations: listeners are more likely to make a high-level tone judgment after hearing an F0 lowered speech context and a low-level tone judgment after hearing an F0 raised speech context. However, listeners, irrespective of non-musicians or musicians, did not show such a pattern in non-speech or music context conditions (Figure 4).

Regarding to IR, the Group factor did not show a main effect [*F*(1,38) = 0.02, *p* = 0.890, η^2^ < 0.001] or any interactions with Context or Shift factors (*p*s > 0.2), and there was not a three-way interaction among the between and within-subject factors (*p* = 0.211). Echoing the results observed in the analysis of PH, such a pattern indicated that the musical training induced familiarity in music contexts did not facilitate listeners to take advantage of non-speech or music contexts for subsequent lexical tone normalization.

### Explorative Analyses on the Effect of Piano Learning Experience on Extrinsic Normalization

A primary aim of this study was to compare musicians with non-musicians on their tone normalization performance following contexts with different familiarity. Surprisingly, the Group factor was not significant in the above analysis, and it did not show any interaction with other factors. One possible reason for such a result was that the musician group had a diverse musical background (Supplementary Table S1) and not all of them were equally familiar with piano notes used in the music context conditions. Thus, we sought to explore whether musicians with piano learning experiences could better facilitate them to adopt music contexts compared with others who did not learn piano before. As in Supplementary Table S1, 12 musicians reported piano learning experience and seven did not learn piano before the experiment. One participant failed to report her learned instrument and was excluded from the subsequent analysis. Within the 19 musicians, a repeated-measures ANOVA was performed to explore the effects of within-subject factors Context, Shift, and the between-subject factor piano learning experience (with two levels, yes and no, and coded as KnowPiano in the following text) on their PH and IR.

Critical to our interest, KnowPiano factor did not significantly influence musicians’ PH [*F*(1,17) = 2.73, *p* = 0.117, η^2^ = 0.020] or IR [*F*(1,17) = 0.20, *p* = 0.661, η^2^ < 0.001]. KnowPiano factor did not interact with Context or Shift factors, either (all *p*s > 0.3), and there was not a three-way interaction (*p* > 0.1). The Context and Shift factors revealed similar main effects and interactions as the previous section, indicating that musicians, regardless of their piano learning experience, perceived targets following the expectation of contrastive context effect only in speech context conditions. This pattern was also consistent with the finding that musicians as a group did not outperform non-musicians in extrinsic tone normalization. The ANOVA tables are summarized in Supplementary Table S2.

## Discussion

In this study, we sought to clarify the possible interference of familiarity factor in listeners’ speech normalization. The familiarity factor was manipulated on two dimensions with musical materials and musical training experience, respectively. Music is a commonly experienced non-verbal stimulus that has higher familiarity than rarely heard synthesized non-speech. In addition, a group of musicians with sufficient musical training was also included in the experiment, as their familiarity with music context was higher than the non-musician group. Previous studies conflict with each other on whether only speech context can provide valid information for listeners to adopt in mapping ambiguous acoustic signals to determinate linguistic units, possibly because the speech and non-speech contexts not only contrast in their linguistic features but also the listeners’ familiarity with these contexts. According to the hybrid model (Tuller, 2003; Nguyen et al., 2009), the lack of non-speech exemplars in the lower layer due to unfamiliarity may account for the speech context superiority in speech normalization. Here we used a non-linguistic context, music, in addition to the conventionally used synthesized non-speech and speech contexts to probe listeners’ lexical tone normalization in a word identification task. Our result showed that the music context did not trigger the lexical tone normalization process in either group. Musicians performed similarly to non-musicians in the music context. Additionally, musicians with piano training did not show a normalization advantage in the music context composed of piano notes compared with musicians without piano training. Overall, the results indicate that the familiarity factor does not interfere with listeners’ lexical tone normalization.

Sjerps and Smiljanić (2013) and Magnuson et al. (2021) reported that the language familiarity and the talker familiarity did not facilitate the accommodation of speech variabilities (but see Lee et al., 2009 and Kang et al., 2016 for different results). By extending their works to the familiarity with different sounds (i.e., speech, music, triangle waves), the present study also failed to find the familiarity advantage in the lexical tone normalization process. Although familiarity advantage has been reported in several aspects of speech perception, for example, better speaker and word identification in noise (e.g., Johnsrude et al., 2013), familiarity might not directly affect the normalization process. Previous studies came up with different explanations for the absence of familiarity advantage in speech normalization. Sjerps and Smiljanić (2013) suggested that the normalization process occurred at the pre-phonemic level, and the acoustic cues were enough to normalize speech variability. Therefore, language familiarities that mainly differed at the phonological level showed no advantage in their studies. However, this explanation was not supported by the present study since we did not observe a reliable normalization effect in the non-speech and music contexts that provided acoustic cues. Magnuson et al. (2021) suggested that the talker normalization process which computes the talker-specific vocal tract characteristics probably overlaps with the talker recognition process. The talker familiarity advantage emerges only when the talker-specific speech characteristics were processed. That is, only when the listener recognized the identity of the talker, can they retrieve the stored exemplars or other mental representations of that talker. Further studies which explore the time course of taker identification and speech normalization should be conducted to test this hypothesis. Here we attempted to give another potential explanation for the absence of familiarity advantage in the lexical tone normalization process. Apart from more exemplars, the familiarity advantage could be aroused by the improved processing proficiency. The empirical studies supported that familiarity facilitates automatic face recognition and automatic speech prosody perception (Ylinen et al., 2010; Yan et al., 2017). Zhang et al. (2017) asked listeners to perform a Cantonese homophone judgment task while listening to speech or non-speech context in the normalization task. They found that the normalization results in both speech and non-speech contexts were not affected by the simultaneously ongoing secondary task. Although speech normalization probably is a cognitive-resource-dependent process (Nusbaum and Morin, 1992), Zhang et al. (2017) indicated that extracting information from both speech and non-speech context is automatic. The automatic extraction of the context pitch was almost not affected by familiarity, resulting in comparable results in music context and non-speech context, and between musicians and non-musicians.

The present study compared different contexts (speech, music notes, vs. triangle waves) and different groups of listeners (musicians vs. non-musicians). Neither dimension showed a familiarity advantage in Cantonese tone normalization, indicating that the speech superiority in Cantonese tone normalization is not due to familiarity but much more likely due to the speech-specific information in speech context. A previous study suggested that the richness of linguistic information influences the magnitude of tone normalization. The removal of semantic, phonological, and phonetic information gradually fails to elicit the contrastive context effect (Zhang et al., 2015). Other researchers hypothesized the speech-specific information enclosed in the talker-specific mapping of acoustic patterns onto linguistic units, and such a mapping is critical for tuning speech perception (Joos, 1948). Even the spectrally rotated non-speech that has more speechlike spectrotemporal dynamics could generate stronger normalization effects than the non-speech context without these speechlike properties (Sjerps et al., 2011). All these studies emphasized the importance of speech-specific (or at least speechlike) information in accommodating speech variability. As music notes and triangle waves in the present study contained no speech-specific information (even no speechlike spectrotemporal dynamics), the normalization process did not emerge. The necessity of the speech-specific information indicates that the successful lexical tone normalization process is largely operated via a speech-specific mechanism. It is worth noting that our findings could only conclude that the familiarity did not contribute to the final decision of the tone categorization. Future studies with a fine-grained temporal resolution (e.g., electrophysiological methods) may provide evidence on whether familiarity influences the early stages of normalization processing.

Aside from the speech-specific information, the coherence between context and target is another potential factor that leads to the speech-superiority in the normalization process. Speech context is more coherent with speech targets in many dimensions than music notes and triangle waves. This is partially supported by the congruency effect reported by Zhang et al. (2017). They found that the pitch height estimation of the non-speech target was better with the non-speech than speech context, and the lexical tone perception of the speech target was better with the speech than the non-speech context. Although the experimental design of the present study cannot tease apart the context-target coherence and the speech-specific information, the coherence hypothesis to some extent is in line with the domain-specific sound process, which in turn supports the speech-specific normalization process. Further studies which include a music context-music target and triangle wave context - triangle wave target could be ideal to test the context-target coherence hypothesis.

Although the studies from our group (e.g., Zhang et al., 2013, 2017) and other research groups (e.g., Francis et al., 2006) consistently revealed the necessity of the speech-specific information in normalizing Cantonese level tones, the normalization of other linguistic units showed mixed results. Huang and Holt (2009) compared the normalization of Mandarin T1 and T2 in the speech context and the non-speech contexts composed of either sine-wave harmonics or pure tone. They found the statistically comparable normalization effect between speech and two non-speech contexts. However, with an almost similar paradigm, Chen and Peng (2016) found the normalization effect in the speech context but not in the non-speech context (triangle waves). The results for the segmental normalization, for example, vowels, are also complex. Sjerps et al. (2011) found that the non-speech context could hardly help the normalization of vowels but once it shared some speech-like spectrotemporal features, the normalization effect emerged. Zhang K. et al. (2018) reported that although at the group level there was no normalization effect for the non-speech context, around half of the participants did show a contrastive context effect in the non-speech condition. These results suggest that non-speech contexts to some extent affect vowel normalization. The discrepancy between level tones and other speech units might come from the acoustic cues that contribute to their identification (Sjerps et al., 2018). The differentiation of level tones mainly depends on the pitch height and the contextual information is important to tell the relative pitch height. This special feature makes the Cantonese level heavily rely on contextual information. However, more than one cue affects the perception of vowels. The pitch and the formant pattern within the target syllable also contribute to the vowel identification (Johnson, 2005). Consequently, the vowel normalization probably relies less on the context information. Although the information in non-speech context is not as rich as that in speech context, it is enough to trigger successful vowel normalization. It is worth noting that no matter for segments or suprasegments, the normalization effect is salient in speech context and does not always appear in non-speech context. Therefore, although the speech-specific context information might not be indispensable for the normalization of phonemes containing rich acoustic cues (e.g., vowels), the superiority of speech context in the extrinsic normalization largely holds in speech perception.

To our knowledge, there was no study directly testing whether music experience facilitates lexical tone normalization. The present study uniquely and empirically probed into this question by comparing musicians and non-musicians. Musicians who are trained intensively in perceiving the fine pitch differences are expected to form a more precise pitch range reference to estimate the relative pitch height of the target lexical tone, and consequently, show a stronger contrastive context effect than non-musicians. However, musicians in the present study showed no advantage in the lexical tone normalization in the speech context, suggesting that there is almost no positive transfer in the pitch encoding from the music domain to the linguistic domain. This finding is somewhat in line with previous studies about musicians of tonal language speakers. Although non-tonal language speakers showed the music experience benefit in the lexical tone discrimination and identification (e.g., French speakers in Marie et al., 2011 and English speakers in Alexander et al., 2005), this benefit reduced a lot for tonal language speakers. Wu et al. (2015) and Zhu et al. (2021) reported that Mandarin musicians only showed the increased sensitivity to the within-category differences which was not important for the lexical tone categorization. Cooper and Wang (2012) found that either tone-language or music experience facilitated the lexical tone identification, but that the combination of two did not lead to better results than either experience alone. By investigating the extrinsic normalization of lexical tones, the present study extended these findings and showed that musicians of tonal language speakers had almost no observable advantage in identifying the relative pitch height in the linguistic domain. Zhang (2020) and Zhang and Peng (2021) found that the normalization process is largely implemented at the phonetic and phonological processing stages. Wu et al. (2015) and Zhu et al. (2021) suggested that acoustic processing was reliably enhanced even with limited musical training (4–5 years of amateur learning), but the musical training did not benefit the phonological processing. This might account for why musicians did not outperform non-musicians in the normalization task. Besides, musicians showed no normalization advantage in the non-verbal context (i.e., piano notes and triangle waves) as well. Although musicians have a more precise encoding of pitch information, the pitch extracted from non-speech contexts is still not enough for them to establish an effective talker-specific reference, indicating that additional speech-specific information is necessary for the successful normalization process (Zhang et al., 2015). Shao and Zhang (2018), Zhang C. et al. (2018), and Liu et al. (2021) consistently reported that amusics of tonal language speakers who are impaired in music pitch perception also show impaired lexical tone normalization, indicating a negative transfer from music pitch processing to linguistic pitch processing. By investigating the musicians’ lexical tone normalization, the present study, however, failed to find a positive transfer from the music domain to the linguistic domain. It is reported that music and language share the similar acoustic parameters and the similar process at the lower level (i.e., the acoustic level), which leads to the observed positive/negative transfer across two domains (Patel, 2013). The accurate perception of the acoustic differences is the basis for the successful processing at the higher level (i.e., the phonological identification). Amusics who are impaired at perceiving the fine acoustic differences are less likely successful at the lexical tone normalization which is largely implemented at the phonetic and phonological level, especially when the demand for pitch sensitivity is high (Wang and Peng, 2014). Meanwhile, successful phonological identification requires acoustic differentiation ability, but the basic acuity shared by normal tonal language speakers (non-amusics) is enough (Cooper and Wang, 2012). This might be the reason why Cantonese musicians who have higher ability at telling fine acoustic differences performed equally well as Cantonese non-musicians in the lexical tone normalization.

## Conclusion

In this study, to evaluate whether the familiarity factor mediates the speech superiority in lexical tone normalization, we compared musicians’ and non-musicians’ perception of Cantonese level tones in speech, music, and non-speech contexts. We found that despite two groups of participants showed clear contrastive context effect in speech context conditions, neither group showed such an effect in non-speech or music context conditions. The familiarity of music could not increase its usefulness in listeners’ speech normalization, even it was longitudinally learned and practiced by listeners. Thus, our findings add more evidence to support the speech-specific mechanism in explaining the speech normalization process. The present study also found that even though musicians have the sophisticated pitch perception ability in music, their music experience does not boost the pitch height estimation in the linguistic domain as revealed by the comparable extrinsic normalization results of musicians and non-musicians.

## Data Availability Statement

The data that support the findings of the current study are available upon reasonable request.

## Ethics Statement

The studies involving human participants were reviewed and approved by Human Subjects Ethics Sub-committee of The Hong Kong Polytechnic University. The participants provided their written informed consent to participate in this study.

## Author Contributions

RT and GP conceived and designed the experiment. RT and KZ implemented the experiment, and collected and analyzed the data. All the authors interpreted the data, wrote the manuscript, and approved the submitted version.

## Conflict of Interest

The authors declare that the research was conducted in the absence of any commercial or financial relationships that could be construed as a potential conflict of interest.

## Publisher’s Note

All claims expressed in this article are solely those of the authors and do not necessarily represent those of their affiliated organizations, or those of the publisher, the editors and the reviewers. Any product that may be evaluated in this article, or claim that may be made by its manufacturer, is not guaranteed or endorsed by the publisher.

## Supplementary Material

Table 1.DOCX Supplementary analysis, supplementary figures, and supplementary tables.

Audio 1–8.WAV Sound files of speech contexts and targets produced by four talkers (MH/ML/FH/FL).
