# Supplementary material for: Music Does Not Facilitate Lexical Tone Normalization: A Speech-Specific Perceptual Process
Source: Front Psychol. 2021 Oct 22;12:717110. doi: 10.3389/fpsyg.2021.717110 (PMC8585521; doi:10.3389/fpsyg.2021.717110)
Supplement: Table 1.DOCX — Supplementary analysis, supplementary figures, and supplementary tables. [file Table_1.DOCX]

**Supplementary material**

Supplementary analysis

In addition to the ANOVA procedure described in section 3.1, we also performed a non-parametric Aligned Ranked Transformed (ART) ANOVA (Wobbrock et al., 2011), which revealed similar results to that specified in the main text. The ART procedure relies on a preprocessing step to align data before applying averaged ranks. Then the data can be entered to point common ANOVA procedures to get statistic values for each main effect and interaction (Wobbrock et al., 2011). The ART ANOVA procedure has the advantage over other non-parametric ANOVA procedures (Kruskal-Wallis, Wilcoxon, Friedman tests, etc.) for its capability of handling repeated measures design with multiple factors and interactions (Wobbrock et al., 2011), which is crucial to our study. The ART ANVOA procedure is realized in R (version 4.0.5) with package ARTool (version 0.11.0).

The ART ANOVA reported that there was a main effect of Choice (F(2, 1786) = 287.85, *p* < 0.001). Post-hoc analysis revealed that response rates of three choices followed high < low < middle-level tone pattern (*p*s < 0.01). The interaction between Context and Choice was also significant (F(6, 1768) = 33.55, *p* < 0.001), and simple-effect analysis revealed the response rates of speech context conditions were higher for middle-level tone response, but lower for low-level tone response than isolated condition (*p*s < 0.001). The difference of high-level tone response rates between speech and isolated context conditions was not significant (*p* = 0.10), probably because loss of power due to non-parametric procedure. Interestingly, the ART ANOVA revealed an interaction between Choice and Group (F(2, 1768) = 5.25, *p* = 0.005). Simple effect analysis showed that musicians did not differ from nonmusicians on response rates of high (19.8% ± 12.6% vs 15% ± 14.8%, F(1, 638) = 2.25, *p* = 0.13) or middle (52.1% ± 14.4% vs 52.8% ± 10.7%, F(1, 638) = 0.03, *p* = 0.86) -level tone choices, but musicians gave less low-level tone choice (28.1% ± 8.6% vs 32.2% ± 12.2%, F(1, 638) = 6.44, *p* = 0.01) responses.

The ART ANOVA results were similar to the repeated-measures ANOVA performed in the main text, except that it revealed a difference between low-level tone response rates between two groups. It seems that musicians tended to make a more balanced wrong choice than nonmusicians, e.g., the musicians' response rate of high- and low-level tones were closer to each other (19.8% and 28.1%), while the nonmusicians were more biased to low-level tone responses (15.0% and 32.2%). Nonetheless, there was not a difference between musicians and nonmusicians in their response rates on the correct choice, e.g., the middle-level tone. This echoed the results in the main text that musical experience did not facilitate musicians to adopt contextual information to regulate the desired responses.

Supplementary figures

**
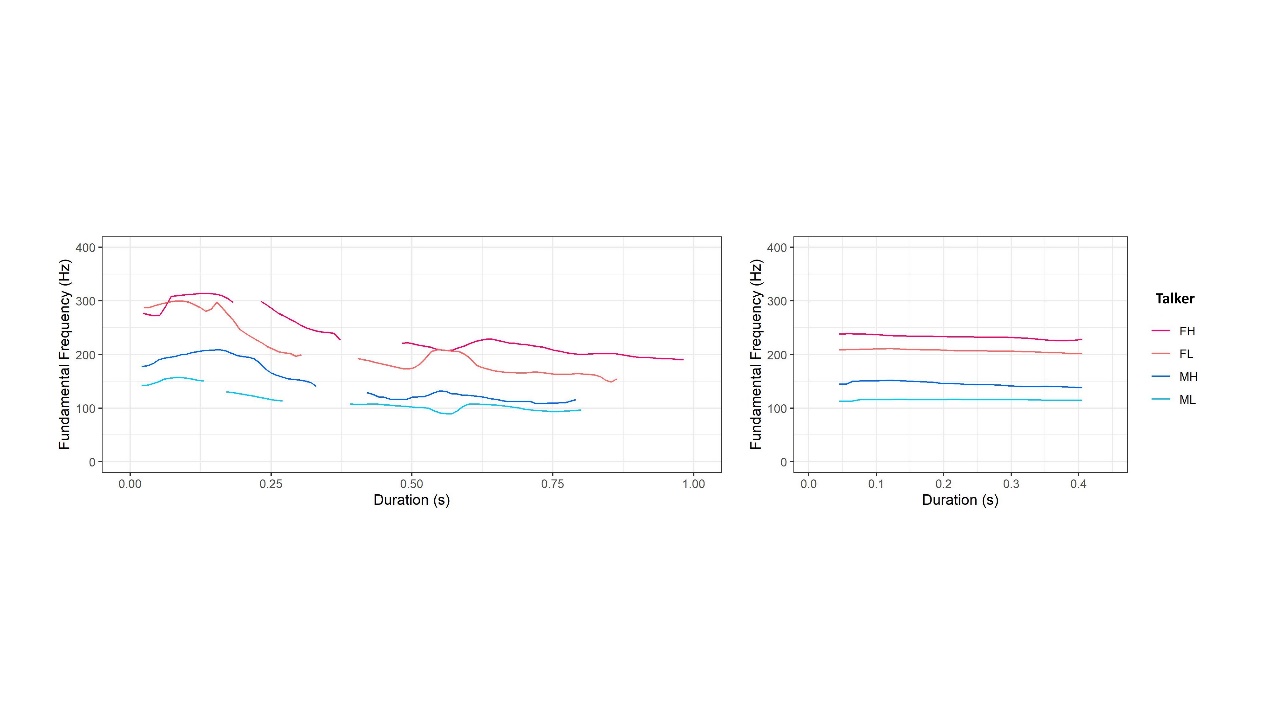
**

Figure S1: Fundamental Frequency (F0) trajectories of speech contexts and targets produced by four talkers. FH: female talker with high pitch; FL: female talker with low pitch; MH: male talker with high pitch; ML: male talker with low pitch. See the supplementary material for corresponding sound files.


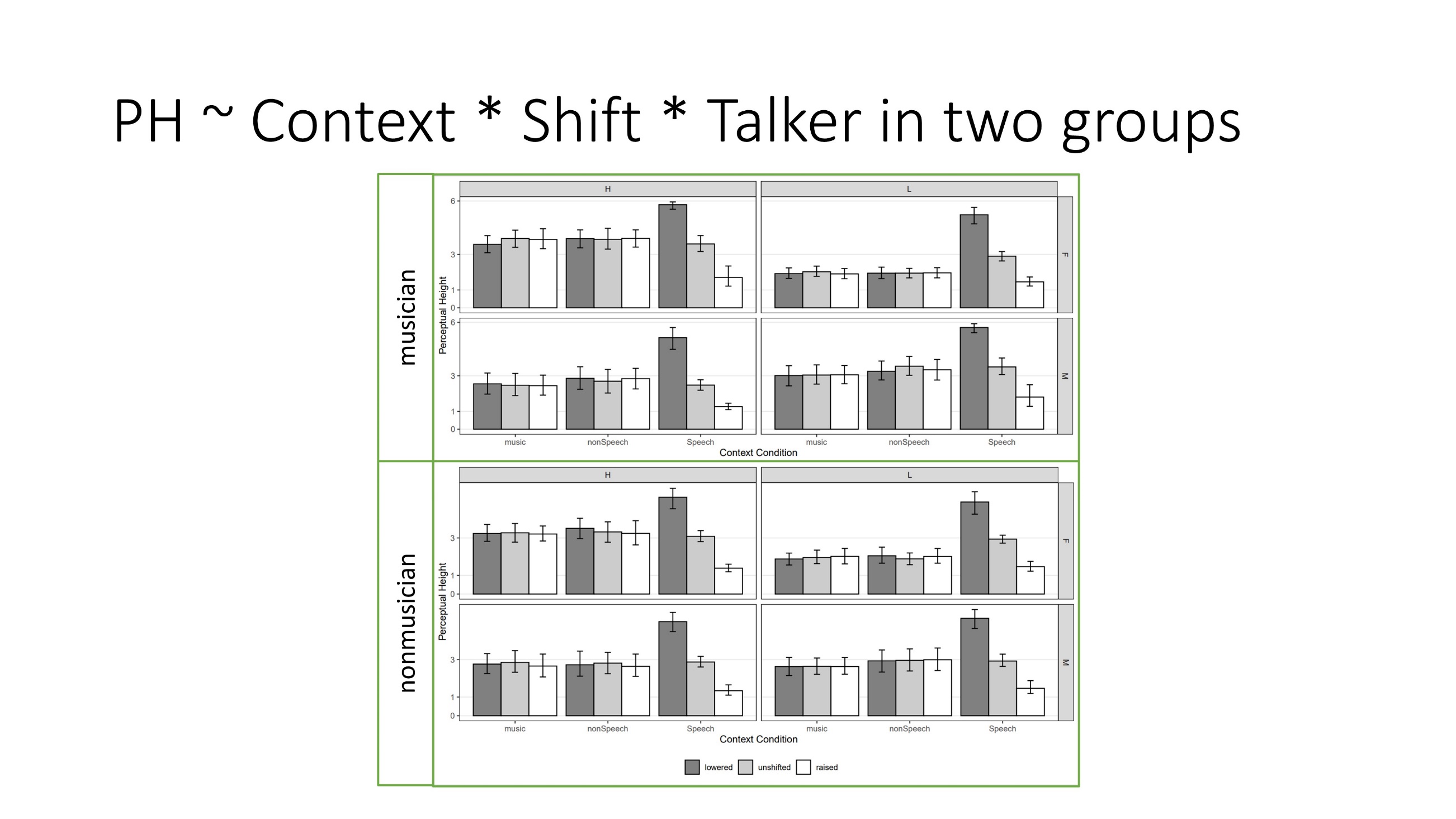


Figure S2: The perceptual height of the targets following three contexts produced by four talkers with F0 manipulations in two groups. Only the speech context elicited evident contrastive contextual effect in both groups and across talkers. The music and nonspeech contexts did not elicit a contrastive contextual effect in either group. F: female; M: male; H: high pitch range; L: low pitch range. Error bars represent 95% confidence intervals.


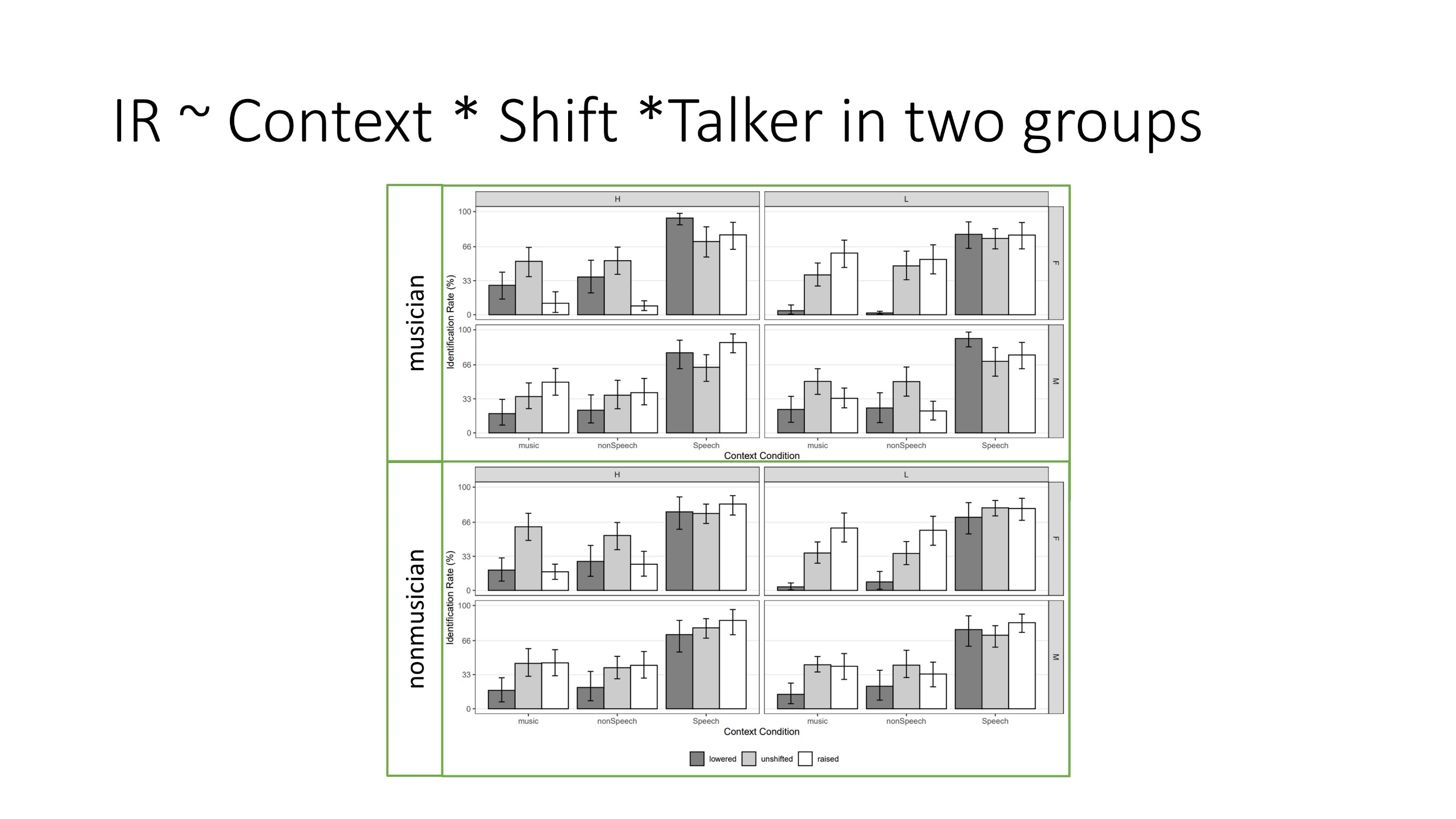


Figure S3: The identification rate of the targets produced by four talkers and perceived as expected responses according to the contrastive context effect in two groups. Targets in the speech context conditions elicited high identification rate with three kinds of F0 manipulation, indicating an evident contrastive context effect and lexical tone normalization process. The music and nonspeech contexts did not elicit lexical tone normalization process. F: female; M: male; H: high pitch range; L: low pitch range. Error bars represent 95% confidence intervals.

Supplementary Tables

Table S1: Characteristics of musician participants.

| Participant no. | Age | Gender | Handedness | Instrument | Years of experience | Age of onset | |  |
| --- | --- | --- | --- | --- | --- | --- | --- | --- |
| 1 | 19.08 | F | R | not reported | 13 | | 6 | |
| 2 | 21.88 | M | R | Piano Trumpet Harmonica Percussion | 15 | | 6 | |
| 3 | 21.84 | M | R | Piano Vocal | 7 | | 14 | |
| 4 | 18.91 | F | R | Piano | 10 | | 6 | |
| 5 | 29.68 | M | R | Piano Contrabass | 13 | | 4 | |
| 6 | 23.94 | M | R | Piano | 16 | | 4 | |
| 7 | 31.07 | M | R | Piano Drum | 20 | | 11 | |
| 8 | 19.1 | F | R | Piano Flute Vocal Chorus | 13 | | 4 | |
| 9 | 18.81 | F | R | Piano | 9 | | 5 | |
| 10 | 30.86 | F | R | Huqin Piano | 24 | | 6 | |
| 11 | 20.2 | F | R | Zhongruan Guitar | 12 | | 9 | |
| 12 | 20.6 | M | R | Guitar | 7 | | 14 | |
| 13 | 19.35 | M | R | Erhu | 12 | | 6 | |
| 14 | 20.98 | F | R | Piano | 14 | | 6 | |
| 15 | 20.08 | F | R | Erhu | 8 | | 13 | |
| 16 | 20.61 | M | R | Guitar | 7 | | 14 | |
| 17 | 26.74 | M | R | Sanxian Pipa | 14 | | 12 | |
| 18 | 28.89 | M | R | Violin Viola | 18 | | 10 | |
| 19 | 27.55 | F | R | Percussion Violin Piano | 19 | | 8 | |
| 20 | 31.34 | F | R | Piano Zheng | 10 | | 9 | |

Table S2: Summary of ANOVA table. The ANOVAs were done to explore whether piano learning experience (coded as KnowPiano) had a significant influence on musicians’ tone normalization performance. See section 3.3 in the main text.

| Dependent variable | Effect | df | MSE | F | η^2^ | p.value |
| --- | --- | --- | --- | --- | --- | --- |
| Perceptual Height | |  |  |  |  |  |
|  | KnowPiano | 1, 17 | 6.01 | 2.73 | .020 | .117 |
|  | Context | 1.85, 31.41 | 3.36 | 6.51 ** | .047 | .005 |
|  | KnowPiano:Context | 1.85, 31.41 | 3.36 | 0.63 | .005 | .527 |
|  | Shift | 1.33, 22.55 | 1.23 | 111.18 *** | .181 | <.001 |
|  | KnowPiano:Shift | 1.33, 22.55 | 1.23 | 1.18 | .002 | .305 |
|  | Context:Shift | 1.62, 27.57 | 1.87 | 127.50 *** | .322 | <.001 |
|  | KnowPiano:Context:Shift | 1.62, 27.57 | 1.87 | 2.06 | .008 | .154 |
| Identification Rate | |  |  |  |  |  |
|  | KnowPiano | 1, 17 | 0.14 | 0.20 | <.001 | .661 |
|  | Context | 1.09, 18.47 | 0.26 | 101.76 *** | .353 | <.001 |
|  | KnowPiano:Context | 1.09, 18.47 | 0.26 | 1.02 | .005 | .333 |
|  | Shift | 1.85, 31.47 | 0.19 | 4.86 * | .032 | .016 |
|  | KnowPiano:Shift | 1.85, 31.47 | 0.19 | 1.00 | .007 | .373 |
|  | Context:Shift | 3.69, 62.69 | 0.10 | 13.23 *** | .089 | <.001 |
|  | KnowPiano:Context:Shift | 3.69, 62.69 | 0.10 | 0.80 | .006 | .523 |

*: *p* < 0.05; **: *p* < 0.01; ***: *p* < 0.001.

Reference:

Wobbrock, J. O., Findlater, L., Gergle, D., & Higgins, J. J. (2011). The aligned rank transform for nonparametric factorial analyses using only anova procedures. *Proceedings of the SIGCHI Conference on Human Factors in Computing Systems*, 143–146. <https://doi.org/10.1145/1978942.1978963>

Kay M, Elkin L, Higgins J, Wobbrock J (2021). ARTool: Aligned Rank Transform for Nonparametric Factorial ANOVAs. doi: 10.5281/zenodo.594511
